# Low physical activity among middle-aged type-2 diabetic outpatients of two peripheral hospitals in Bangladesh

**DOI:** 10.1371/journal.pone.0284392

**Published:** 2023-04-13

**Authors:** Rajib Mondal, Palash Chandra Banik, Mohammad Mostafa Zaman

**Affiliations:** 1 Department of Public Health, Hamdard University Bangladesh, Gazaria, Munshiganj, Bangladesh; 2 Department of Noncommunicable Diseases, Bangladesh University of Health Sciences, Dhaka, Bangladesh; 3 Ekhlaspur Center of Health, Chandpur, Bangladesh; Texas State University, UNITED STATES

## Abstract

Physical activity (PA) is an important lifestyle recommendation for managing type-2 diabetes mellitus (T2DM). However, low PA among them is a global public health concern, including Bangladesh. We aimed to investigate the prevalence of low PA levels and its associated sociodemographic factors particularly among middle-aged T2DM subjects, which is quite limited globally and unknown in Bangladesh. In this cross-sectional study, we conveniently recruited 356 T2DM subjects (aged 40–60 years) from outpatient departments of the corresponding diabetic hospitals from Pirojpur and Dinajpur, the southern and northern districts, respectively. The primary outcome was low PA (via metabolic equivalents <600), using the Global Physical Activity Questionnaire. Univariable and multivariable binary logistic regression analyses were used to identify the factors associated with low PA. Among the participants (mean age 51.0±6.9 years), men and women were with almost equal proportions (48.9% and 51.1%, respectively). The prevalence (95% Confidence Intervals [CI]) of low PA was 34.8% (29.9–39.7). The median sitting or reclining time was 6 hours on a typical day. The odds (OR [95% CI]; *P*) of low PA was found to be significantly higher in respondents with primary or no education compared to the above-primary level, in unadjusted (1.6 [1.1–2.6]; 0.029) and adjusted (2.0 [1.1–3.7]; 0.028) associations both. In conclusion, over one-third of the middle-aged study subjects had a low PA level, which was associated with education. There is a high demand for designing and implementing PA enhancing interventions among them.

## Introduction

Regular physical activity (PA) is essential to prevent and manage a number of chronic diseases, including type-2 diabetes mellitus (T2DM). The World Health Organization (WHO) recommends an adult individual to perform at least 150–300 minutes of moderate- or 75–150 minutes of vigorous-intensity PA, or an equivalent combination of moderate- to vigorous-intensity PA in a typical week. However, one-quarter of the individuals are not reaching this recommended level [1]. Currently, this low PA (in other words, ’physical inactivity’ or ’insufficient PA’) is being considered as a global public health concern [1,2]. Worldwide, statistics reveals that around 1.6 million annual deaths are attributed to low PA [2].

T2DM is substantially related to several lifestyle-related factors like PA [3]. Study with middle-aged individuals discloses a sound glycemic control and a low risk of developing T2DM when they engage in moderate levels of PA even [4]. To attain a better management of blood sugar levels in T2DM subjects, participating in regular PA and decreasing prolonged inactive practices is highly recommended [5]. However, over 60% of the T2DM subjects in the United States remain with low PA [6], whereas it is 15–74% throughout European countries [7–10] and 43–51% in Australia [11]. In neighboring countries, it is 20% of urban Nepalese [12], 14% of Sri Lankans [13], and 56% and 74% of Indians from Andhra Pradesh [14] and Tamil Nādu [15], respectively.

In Bangladesh, a recent study in Dhaka shows that nearly one-third (31.2%) of T2DM subjects have low PA, which is significantly associated with their occupation and family income [16]. Around a decade ago, a similar study from this city reported low PA with a much higher proportion (74%) among T2DM subjects [17]. These sporadic studies indicate that exploring PA levels among T2DM subjects is an inadequately studied area in this country. Moreover, the information related to PA situation among the middle-aged T2DM population is still unknown, especially in peripheral areas. We aimed to investigate the prevalence of low PA and its associated sociodemographic factors in middle-aged T2DM subjects in this country’s selected southern and northern districts to gain insight into the levels of PA among them in such areas away from the capital city.

## Materials and methods

### Study design, setting, population, and sampling

This cross-sectional study was carried out in 2017 among 356 T2DM subjects in Alhaz Asmat Ali Khan Diabetic Hospital, Pirojpur district, and Dinajpur Diabetic Hospital, Dinajpur district. Both men and women aged 40–60 years with intact functional ability for PA were enrolled from the outpatient departments (OPD), using a convenience sampling method. Pirojpur and Dinajpur districts are located in the southern and northern peripheral parts of Bangladesh, respectively. The hospitals are the branches of the Diabetic Association of Bangladesh (BADAS), a leading tertiary-level healthcare organization for the prevention, management, control, and rehabilitation of diabetes.

### Outcomes

This study’s primary and secondary outcomes were the prevalence of low (or insufficient) PA levels and its associated sociodemographic factors, respectively.

### Data collection instrument and technique

We adopted the ‘Global Physical Activity Questionnaire (GPAQ)’ version-2 [18] and translated it into the Bengali language (following the WHO STEPS-wise noncommunicable disease [NCD] risk factor survey Bangladesh 2010 [19]) for a better understanding of the questions. In face-to-face interviews, the respondents were asked about their sociodemographic and PA (frequency in days in a typical week and time spent in minutes/hours)-related questions, and the self-reported responses were recorded. The sociodemographic part included sex, age, education, occupation, and monthly family income-related information, and the GPAQ collects information on PA participation (at least for 10 minutes) in the following three main domains- i) activity at work, ii) travel to and from places, and iii) recreational activities. The ’activity at work’ and ’recreational activities’ domains comprise both moderate-intensity (e.g., moderate work, cycling, swimming, playing volleyball, etc.) and vigorous-intensity PA (e.g., vigorous work, playing football, etc.), whereas the ’travel to and from places’ domain comprises only moderate-intensity PA (e.g., traveling to/from workplace, marketplace, etc. by walking/cycling, including diabetic patient’s routine walking for this study). Moreover, an additional ’sedentary behavior’ domain records sedentary spending sitting or reclining times.

### PA assessment and measurement method

PA was assessed in metabolic equivalents (METs) min/week using the following equations [18]-

1. Work-related PA METs min/week = (Number of days used to perform vigorous-intensity PA × Duration in min × 8) + (Number of days used to perform moderate-intensity PA × Duration in min × 4).
2. Transport-related PA METs min/week = Number of days used to perform transport-related PA × Duration in min × 4.
3. Recreational PA METs min/week = (Number of days used to perform vigorous-intensity PA × Duration in min × 8) + (Number of days used to perform moderate-intensity PA × Duration in min × 4); and
4. Total METs min/week = (work + transport + recreational)-related PA METs min/week

Based on cumulative METs/week, the respondent’s PA levels are defined as low (METs <600), moderate (METs 600–3,000), and high (METs >3,000) [19,20].

### Statistical analyses

SPSS software (IBM Corp. Released 2013. IBM SPSS Statistics for Windows, Version 21.0. Armonk, NY: IBM Corp.) was used for statistical analyses. Descriptive statistics were used for sociodemographic and PA-related variables. Independent Sample *t*-test, Mann-Whitney U test, and Chi-square test were used to see the PA differences in men and women. Univariable and multivariable binary logistic regression analyses were used to assess the associations of sociodemographic factors with low PA, computing the unadjusted and adjusted odds ratios (with 95% confidence intervals [CI]), respectively. In our multivariable models adjusted for all five dichotomized sociodemographic factors (since these are the most vital sociodemographic factors usually highly influential to the main outcome and also most of these were found to be correlated to each other in our study) simultaneously as well as all possible pairs of these factors (e.g. sex and age, sex and education, and so on), we considered moderation analyses to explain the interaction terms among these factors whether these modify the low PA outcomes. Odds ratios (OR) were calculated by exponentiating beta estimate, and 95% CI were calculated exponentiating beta ± 1.96 times standard error of beta estimates. *P*<0.05 was considered the statistical significance.

### Ethical statements

This paper has been derived from an unpublished thesis work [21]. The primary thesis research was conducted following the Declaration of Helsinki (as revised in 2013) as a statement of ethical principles for medical research involving human subjects, subject to anonymity. Bangladesh Medical and Research Council guideline was also followed accordingly. Ethical clearance was taken from the Ethical Review Committee of Bangladesh University of Health Sciences (Identification no. BUHS/BIO/EA/17/82). Written informed consent was taken from each respondent.

## Results

### Population characteristics

Of all (mean age 51.0±6.9 years), 205 (58%) were from the Dinajpur district and the remaining were from Pirojpur, men and women were almost equal (48.9% and 51.1%, respectively), not shown in the table. Most of them were 55 years old and above (41.0%), with up to primary level (grade 5) of education (40.7%), homemakers (46.6%), and with monthly family income of 30,000 and above in Bangladeshi Taka (currency), detailed in the Table 1.

**Table 1:** Population characteristics and GPAQ domain-wise physical activity behaviors.

Variables	Total (*n* = 356)	Men (*n* = 174)	Women (*n* = 182)	*P*
	*n* (%)			
**Socio-demographics**				
**Age *(in years)***				
Below 45	76 (21.3)	32 (18.4)	44 (24.2)	
45–49	51 (14.3)	24 (13.8)	27 (14.8)	0.509*
50–54	83 (23.3)	41 (23.6)	42 (23.1)	
55 and above	146 (41.0)	77 (44.3)	69 (37.9)	
**Level of education**				
No formal education	45 (12.6)	10 (5.7)	35 (19.2)	
Up to primary	145 (40.7)	55 (31.6)	90 (49.5)	<0.001*
Up to secondary	73 (20.5)	36 (20.7)	37 (20.5)	
Higher secondary and above	90 (26.1)	73 (42.0)	20 (11.0)	
**Occupation**				
Employed	67 (18.8)	56 (32.2)	11 (6.0)	
Business	73 (20.5)	72 (41.4)	1 (0.5)	<0.001*
Homemaker	166 (46.6)	0 (0)	166 (91.2)	
OthersѰ	50 (14.0)	46 (26.4)	4 (2.2)	
**Monthly family income**, *in BDT*				
Mean±Standard deviation	30,331±11,558	33,914±13,386	26,907±8,162	<0.001*
Below 30,000	149 (41.9)	47 (27.0)	102 (56.0)	<0.001*
30,000 and above	207(58.1)	127 (73.0)	80 (44.0)	
**Physical activity behaviors**				
Vigorous-intensity physical work	18 (5.1)	11 (6.3)	7 (3.8)	0.287*
Moderate-intensity physical work	76 (21.3)	45 (25.9)	31 (17.0)	0.042*
Traveling by walking	277 (78.2)	146 (83.9)	131 (72.0)	0.007*
	**mean±standard deviation**			
**Work-related physical activity**				
Vigorous intensity working days in a week	3.7±1.7	3.0±1.5	4.7±1.6	0.034**ǂ**
Vigorous intensity working duration in a day (in minutes)	131±89	100±36	180±125	0.144**ǂ**
Moderate intensity working days in a week	4.8±2.0	4.8±1.8	4.9±2.3	0.921**ǂ**
Moderate intensity working duration in a day (in minutes)	107±90	121±90	87±87	0.107**ǂ**
**Transport-related physical activity**				
Traveling days in a week	6.0±1.6	5.9±1.7	6.0±1.6	0.517**ǂ**
Traveling duration in a day *(in minutes)*	35±19	34±19	36±19	0.401**ǂ**
**Sedentary hrs./day**, *Median (IQR)*	6.0 (5.0–8.0)	6.0 (5.0–10.0)	5.5 (5.0–8.0)	0.105‡
**Total METs-mins/week**, *Median (IQR)*	840 (255–1400)	840 (480–1680)	840 (150–1260)	0.020‡

GPAQ = Global Physical Activity Questionnaire; BDT = Bangladeshi Taka (currency); IQR = Interquartile range; METs = Metabolic equivalents.

Primary, secondary and higher secondary levels of education imply grade-5, grade-10 and grade-12, respectively.

^Ѱ^Occupation ‘others’ included farmer, day laborer, retired, unemployed etc.

Transport-related physical activity included traveling to/from workplace, marketplace, etc., by walking, including diabetic patient’s routine walking.

*Chi-square test or independent Sample *t*-test; and

^**‡**^Mann-Whitney U test.

### GPAQ domain-wise PA behaviors

The Table 1 also illustrates GPAQ domain-wise PA behaviors. One in 5 (21.3%) was engaged in work-related moderate-intensity PA, wherein men reported marginally significantly higher (p = 0.052). Nearly 4 in 5 (78.2%) were involved in transport-related PA (e.g., traveling by walking), with significantly higher in men (P = 0.007). However, recreation-related vigorous-intensity PA was found to be highly negligible- only 2 (0.6%) of them (men) reported playing football once a week for a median duration of 120 minutes. Also, recreation-related moderate-intensity PA was nil. The sedentary spending median time in a typical day was noticeably high (6 hours) among them. Moreover, the median of PA METs/week was 840, which was significantly higher in men (*P* = 0.020).

### Low and other levels of PA

When categorizing the cumulative METs, the prevalence of low PA was found at 34.8%. While moderate and high PA were found at 55.1% and 10.1%, respectively. PA levels were significantly differed by level of education and monthly family income (Table 2). Moreover, the low PA was significantly higher in respondents from Pirojpur (41.7% vs. 29.8%; *P* = 0.019) than that of Dinajpur (not shown in table). Further exploring the proportions of low PA and the associated factors, we used dichotomous sociodemographic variables considering to meet the sufficient number of sub-samples in each domain.

**Table 2:** Prevalence of low, moderate, and high physical activity levels by sociodemographic characteristics (*n* = 356).

Variables	Low PA	Moderate PA	High PA	*P*
	% (95% Confidence Interval)			
**Sex**				
***Overall***	***34*.*8 (29*.*9–39*.*7)***	***55*.*1 (49*.*9–60*.*3)***	***10*.*1 (7*.*0–13*.*2)***	0.096
Men	31.0 (24.1–37.9)	55.7 (48.3–63.1)	13.2 (8.2–18.2)	
Women	38.5 (31.4–45.6)	54.4 (47.2–61.6)	7.1 (3.4–10.8)	
**Age *(in years)***				
Below 50	31.5 (23.4–39.6)	62.2 (53.8–70.6)	6.3 (2.1–10.5)	0.071
50 and above	36.7 (30.5–42.9)	51.1 (44.6–57.6)	12.2 (8.0–16.4)	
**Level of education**				
Primary or no education	40.0 (33.0–47.0)	45.3 (38.2–52.4)	14.7 (9.7–19.7)	<0.001
Above primary level	28.9 (22.0–35.8)	66.3 (59.1–73.5)	4.8 (1.5–8.1)	
**Occupation**				
Employed	32.8 (21.6–44.0)	62.7 (51.1–74.3)	4.5 (0.5–9.5)	0.169
OthersѰ	35.3 (29.8–40.8)	53.3 (47.5–59.1)	11.4 (7.7–15.1)	
**Monthly family income**, *in BDT*				
Below 30,000	34.9 (27.2–42.6)	47.0 (39.0–55.0)	18.1 (11.9–24.3)	<0.001
30,000 and above	34.8 (28.3–41.3)	60.9 (54.3–67.5)	4.3 (1.5–7.1)	

Low, moderate, and high PA levels were defined when METs/week were <600, 600–3,000, and >3,000, respectively.

A Chi-square test was done.

Primary level of education implies grade-5.

^Ѱ^Occupation ‘others’ included homemaker, businessman, farmer, day laborer, retired, unemployed, etc.

BDT = Bangladeshi Taka (currency).

### Associated factors of low PA

The low PA was slightly higher in women aged 50 years and above and homemakers than their counterparts, although the differences were nonsignificant. However, it was found to be significantly higher (*P*<0.029) in respondents with primary or no education than that of above the primary level (Table 2). The odds (OR [95% CI]; *P*) of low PA were found to be significantly higher in respondents with primary or no education compared to the above primary-level educated ones, in both unadjusted (1.6 [1.1–2.6]; 0.029) and adjusted (2.0 [1.1–3.7]; 0.028) binary logistic regression analyses. No further associations were found significant in this study (Table 3). And, there was no significant interaction effect among the dichotomized sociodemographic factors (neither all factors simultaneously [Table 3] nor the different pairs of factors [not shown in table]) on low PA in our study.

**Table 3:** Crude and adjusted odds ratios of low physical activity (<600 metabolic equivalents) by sociodemographic factors (*n* = 356).

Independent variables	Low PA		Unadjusted	§Adjusted	
	Yes (*n*)	No (*n*)	OR (95% CI); *P*	OR (95% CI); *P*	Interaction *P*
**Sex**					
Men	54	120	Reference	Reference	
Women	70	112	1.4 (0.9–2.2); 0.142	1.4 (0.8–2.1); 0.213	
**Age *(in years)***					
Below 50	40	87	Reference	Reference	
50 and above	84	145	1.3 (0.8–2.0); 0.326	1.1 (0.7–1.8); 0.738	
**Level of education**					0.637
Above primary	48	118	Reference	Reference	
Primary or no education	76	114	**1.6 (1.1–2.6); 0.029**	**2.0 (1.1–3.7); 0.028**	
**Occupation**					
Employed	22	45	Reference	Reference	
OthersѰ	102	187	0.8 (0.4–1.5); 0.704	0.7 (0.4–1.4); 0.365	
**Monthly family income**, *in BDT*					
Below 30,000	52	97	Reference	Reference	
30,000 and above	72	135	1.0 (0.6–1.5); 0.982	1.4 (0.8–2.5); 0.180	

Unadjusted and adjusted binary logistic regression analyses were done.

^§^All dichotomized sociodemographic variables were entered into the adjusted model simultaneously.

Primary level of education implies grade-5.

^Ѱ^Occupation ‘others’ included homemaker, businessman, farmer, day laborer, retired, unemployed, etc.

PA = Physical activity; OR = Odds ratio; CI = Confidence interval; BDT = Bangladeshi Taka (currency).

## Discussion

### Significance and new findings

The present study provides unrevealed information regarding the prevalence of low PA and its associated sociodemographic factors among the middle-aged T2DM population, particularly in Bangladesh. We found that over one-third of the study population remains physically inactive, influenced by a ’no’ or ’low’ level of education. The findings of this study enable us to make sense of how certain aged Bangladeshi diabetic people (with sex-specific distribution) in peripheral areas are physically inactive, leading to a potentially higher burden of the disease. Moreover, the finding shows the difference in low PA levels among the populations of the two selected districts of this country.

### Findings from the Bangladeshi population

In Bangladesh, overall, the country-wide prevalence of low PA among the general population was reported at 12.3–38% in three different STEPS surveys [20,22–24] and 7.5% in another rural community-based study [25]. Moreover, compliant with our findings, the recent Dhaka city-based study reported that around one-third of their T2DM subjects with low PA [16]. The data indicate that many Bangladeshi people remain physically inactive regardless of whether they have diabetes or not and their geographical locations. Moreover, that study found that PA was associated with occupation and family income [16] compared to education in our study. However, we found an essentially contradictory finding to another Dhaka-based study that reported 74% low PA of the diabetic subjects, which might be due to recruiting a significant proportion of subjects with higher age (one-fourth were ˃60 years), overweight and obesity (65%), residing in urban area (capital city) with a very limited space for walking or recreational activity, and also the subjects with diabetic-related complications sought in that study [17].

Furthermore, the situation of recreation-related PA (sports and fitness) has been found to be negligible in our subjects. The reason is that it has not become a part of our society and culture. Sedentary behavior has also been notably higher, even double that of a Dhaka-based recent study [16]. These findings indicate that our study population may spend much sitting or reclining time and may be negligent being engaged in sports and relevant recreation-related activities, which is not suggestible.

### Findings from neighboring country’s diabetic population

We found a better situation of low PA in our study subjects when compared to the same subjects from the adjacent neighboring country India, such as studies from Andhra Pradesh (56%) [14] and Tamilnadu (74%) [15]. However, the low PA situation seems much better in other neighboring countries of this region, such as Nepal (20%) (12) and Sri Lanka (14%) (13), compared with our study. These contradictory findings might happen due to the urban community study settings, differences in sex, level of education and occupation, and self-reported joint pain (14, 15). The possible reasons might be the higher men subjects, a mentionable proportion of subjects aged below 40 years, educated subjects, and relatively laborious occupations such as agriculture in these studies [12,13].

### Limitations and strengths

Subjectively identified PA behavior may cause social desirability bias. A nonrandomized (due to inconvenience in OPD settings) small sample may not meet the population’s representativeness, especially for subgroup comparison. Moreover, the possibility of being enrolled with higher disease complexity in a hospital may reflect lower PA behavior. However, such cases are usually referred to the central BADAS hospital in Dhaka. Despite these issues, our findings will help the policymakers gain insight into the PA situation relatively in a younger group of T2DM subjects from this country’s distinctive peripheral geographical locations and help design and implement necessary PA-enhancing interventions for them.

## Conclusion

Over one-third of the study subjects had a low PA level, which was associated with education. There was notably high sedentary behavior with negligible recreational PA among them. There is a high demand for designing and implementing appropriate PA-enhancing intervention programs among the patients with type-2 diabetes mellitus in Bangladesh.

## Supporting information

S1 File (SAV)
